# Supplementary material for: Thermal detection of single photons using Dirac fermions
Source: Nat Commun. 2026 Mar 12;17:3845. doi: 10.1038/s41467-026-70648-0 (PMC13121805; doi:10.1038/s41467-026-70648-0)
Supplement: Supplementary file 1 — Supplementary Information [file 41467_2026_70648_MOESM1_ESM.pdf]

Supplementary Information: Thermal Detection of Single Photons Using Dirac Fermions

Bevin Huang,[1,*] Ethan G. Arnault,[2,*] Woochan Jung,[3,*] Caleb Fried,[2] B. Jordan Russell,[4,5] Kenji Watanabe,[6] Takashi Taniguchi,[7] Erik A. Henriksen,[4,5] Dirk Englund,[2] Gil-Ho Lee,[3,†] and Kin Chung Fong[8,9,10,11,‡]

[1] *Intelligence Community Postdoctoral Research Fellowship Program, Massachusetts Institute of Technology, Cambridge, MA 02139*
[2] *Department of Electrical Engineering and Computer Science, Massachusetts Institute of Technology, Cambridge, MA 02139*
[3] *Department of Physics, Pohang University of Science and Technology, Pohang 790-784, Republic of Korea*
[4] *Department of Physics, Washington University in St. Louis, St. Louis, MO, USA*
[5] *Center for Quantum Leaps, Washington University in St. Louis, 1 Brookings Dr., St. Louis MO 63130, USA*
[6] *Research Center for Electronic and Optical Materials, National Institute for Materials Science, 1-1 Namiki, Tsukuba 305-0044, Japan*
[7] *Research Center for Materials Nanoarchitectonics, National Institute for Materials Science, 1-1 Namiki, Tsukuba 305-0044, Japan*
[8] *RTX BBN Technologies, Quantum Engineering and Computing Group, Cambridge, Massachusetts 02138, USA*
[9] *Present Address: Quantum Materials and Sensing Institute, Northeastern University, Burlington, MA 01803, USA*
[10] *Present Address: Department of Electrical and Computer Engineering, Northeastern University, Boston, MA 02115, USA*
[11] *Present Address: Department of Physics, Northeastern University, Boston, MA 02115, USA*

(Dated: February 20, 2026)

Device	A	B
JJ width (μm)	1.7	1.7
JJ channel length (nm)	600	600
Graphene layer	1	1
MoRe thickness (nm)	195	195
Contact Type	1D	2D
Graphene Target Width (μm)	4.8	4
Graphene Target Length (μm)	10.6	25.8
$\langle I_s \rangle$ (μA)	3.3	3.3
V_{CNP} (V)	0	-0.15
Bottom hBN thickness (nm)	56	36
Top hBN thickness (nm)	28	51
n_e/V_{gate} (10^{12} cm$^{-2}/V$)	0.22	0.34
Electronic mobility (cm^2/V s)	17000	9100
Mean free path (nm)	313	444
I_c (μA)	3.6	3.38
R_n (Ω)	70	48
I_cR_n (μeV)	252	161
Thouless energy (meV)	1.34	0.91
JJ coupling energy (meV)	7.39	6.9
$\omega_{P0}/2\pi$ (GHz)	199	131
C_{JJ} (fF)	7	15
$Q(I_b = 0)$	0.61	0.59
Δ_s of MoRe (meV)	1.3	

Supplementary Table 1. **List of parameters for measured devices.** V_{CNP} is the gate voltage of the charge neutrality point for the monolayer graphene.

Supplementary Note 1: Determination of hBN and graphite thicknesses

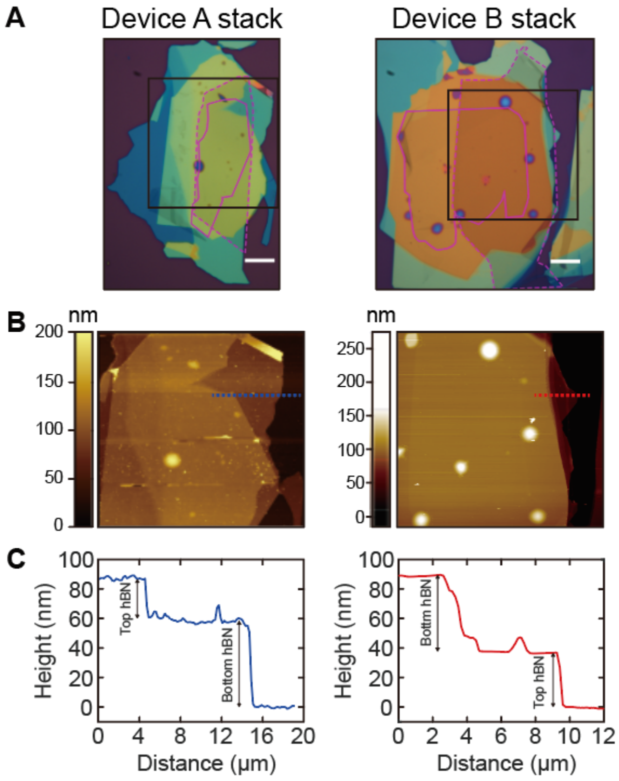

Supplementary Figure 1. **AFM topography of two devices. (A)** Optical microscope images of the stacks for device A (left) and device B (right). The magenta line marks the graphene, while the dashed line marks the graphite. AFM topography was measured in the regions outlined by the black boxes. Scale bar, 10 µm. **(B)** AFM topography of the boxed area in **(A)**, for device A (left) and device B (right). **(C)** Line profiles extracted from **(B)** along the dashed lines: blue (device A, left) and red (device B, right).

* These authors contributed equally to this work
† Corresponding Author: lghman@postech.ac.kr
‡ Corresponding Author: k.fong@northeastern.edu

We determined the thicknesses of the top and bottom hBN layers from AFM topography measurements on the stack, as shown in Supplementary Figure 1. In contrast, the bottom graphite thickness cannot be precisely extracted from AFM due to the limited resolution and the presence of surface impurities on the stack. To address this, we quantified the optical contrast of the graphene region relative to the bare substrate by analyzing the average intensity of the green channel in optical microscope images. This approach follows the method established by Ni [1] and Wang [2], who demonstrated a clear, quantitative correlation between the number of graphene layers on a SiO_2 (≈285 nm)/Si substrate and the green-channel contrast. For example, Wang *et al.* reported that the green contrast increases systematically with thickness: 7.7 %, 14.9 %, 21.6 %, and 27.8 % for one to four graphene layers, respectively. Using this calibration, as shown in Supplementary Figure 2, we assigned the bottom graphite thickness in device A to six layers (≈2 nm, 21.4 %) and in device B to four layers (≈1.33nm, 17.1 %).

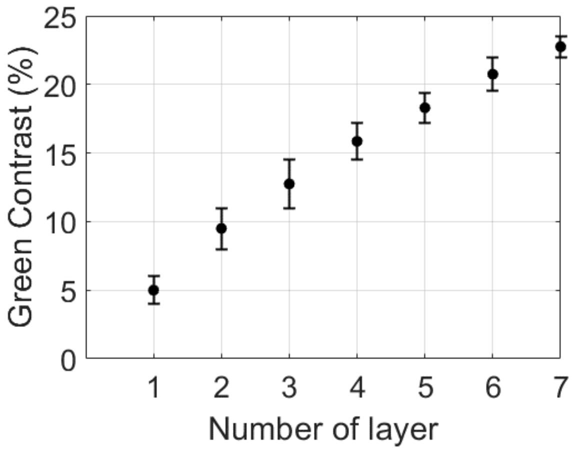

Supplementary Figure 2. Green-channel optical contrast as a function of the number of graphene layers on a SiO_2 (≈285 nm)/Si substrate. The calibration curve is used to assign the thickness of the bottom graphite in devices A and B. Error bars represent the standard deviation of the measured contrast values.

Supplementary Note 2: Characterization of graphene-based Josephson junctions

Supplementary Figure 3 shows the $I-V$ characteristics of the GJJs. Supplementary Figure 3 A and B plots $R_n(V_{\mathrm{gate}})$ with I_b of 4 μA $> \langle I_s \rangle$. Both devices have a sharp, singular charge neutrality point at 0 V and -0.15 V for devices A and B, respectively. This indicates that the charge density is homogeneous in the junction region.

Supplementary Figure 3C and D show the full scan of $R_n(I_b, V_{\mathrm{gate}})$. The robust supercurrent that flows on either side of the charge neutrality point[3–6] is consistent with previous reports in using MoRe to fabricate GJJs [7, 8]. This is attributed to the minimal doping caused by the MoRe on the graphene. Collectively, these measurements indicate high-quality GJJs that can support a wide range of supercurrents for the SPB. Table 1 summarizes the characteristics of the two measured devices.

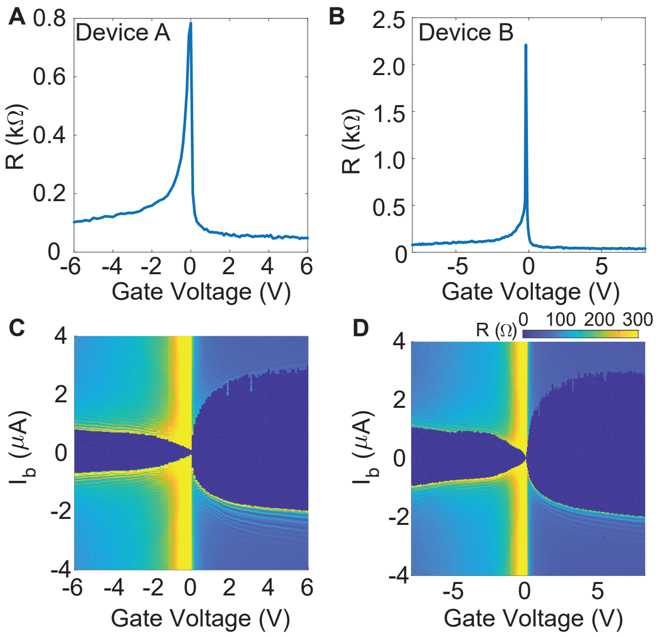

Supplementary Figure 3. **Characterizations of the GJJs. (A)** Device A gate dependence of the normal state resistance R. **(B)** Device B gate dependence. **(C)** Device A bias-gate map. **(D)** Device B bias-gate map.

Supplementary Note 3: Calculating the reflectance and absorption coefficient of hBN-encapsulated graphene

The optical properties of a material depends on its dielectric environment. Similar to superconducting nanowire detectors and transition edge sensors [9–14], we can improve the efficiency of our SPB in the future by optimizing the absorption coefficient of incident photons of the graphene [15–18]. For this report, we calculate the reflectivity of the entire graphene heterostructure, $\mathcal{R}_{\mathrm{hs}}$, and the graphene absorption coefficient, α_{gr}, using the wave-transfer matrix method [10, 19–21]. We find excellent agreement between the measured and calculated ratio of $\mathcal{R}_{\mathrm{hs}}$ on silicon to the graphene heterostructures (Supplementary Figure 2A).

In the wave-transfer matrix method, M_i and $M_{i,j}$ are the matrices describing, respectively, the phase accumulation of light when traverses through the i-th optical layer, and the transmission and reflection at the interface when the light traversing from the j-th to the i-th optical layer [22]:

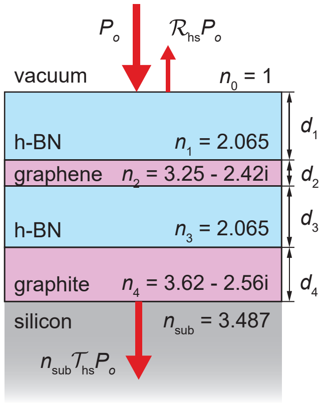

Supplementary Figure 4. **Schematic of the graphene heterostructure in our SPB.** Side view of the heterostructure used to calculate the 1550 nm absorption and reflection values of the graphene SPBs. From top to bottom, the heterostructure consists of alternating h-BN and graphene/graphite layers terminating with a semi-infinite layer of Si. In our calculations, P_0 is the optical power incident from vacuum onto the heterostructure. The power that is transmitted through the heterostructure is $n_{\rm sub}\mathcal{T}_{\rm hs}P_o$ whereas the power that is reflected from the heterostructure is $\mathcal{R}_{\rm hs}P_o$. The red arrows denote the Poynting vector associated with incident, reflected, and transmitted light.

$$M_i = \begin{pmatrix} \exp(-2\pi j n_i d_i/\lambda) & 0 \\ 0 & \exp(2\pi j n_i d_i/\lambda) \end{pmatrix} \quad (1)$$

$$M_{i,j} = \frac{1}{2n_j}\begin{pmatrix} n_j + n_i & n_j - n_i \\ n_j - n_i & n_j + n_i \end{pmatrix} \quad (2)$$

with $\lambda \simeq 1550$ nm being the wavelength of light, n_i the index of refraction, and d_i thicknesses of the i-th layer.

Supplementary Figure 4 depicts the graphene heterostructure in our SPB with the index assignment for each layer. The overall matrix, M, of the hBN/graphene/hBN/graphite heterostructure on a semi-infinite substrate denoted with the subscript "sub" is given by:

$$M = M_{\rm sub,4}\prod_{i=1}^{4} M_i M_{i,i-1} = \begin{pmatrix} m_{11} & m_{12} \\ m_{21} & m_{22} \end{pmatrix} \quad (3)$$

For light of an electric field amplitude in the i-th layer, E_i^β, with $\beta = +(-)$ denoting the light propagation from vacuum to substrate (substrate to vacuum), we have:

$$\begin{pmatrix} E^+_{\rm sub} \\ 0 \end{pmatrix} = M \begin{pmatrix} E^+_0 \\ E^-_0 \end{pmatrix}. \quad (4)$$

$$E^-_0 = -\frac{m_{21}}{m_{22}}E^+_0 \quad (5)$$

$$E^+_{\rm sub} = \left(m_{11} - \frac{m_{12}m_{21}}{m_{22}}\right)E^+_0. \quad (6)$$

Device	d_1 (nm)	d_2 (nm)	d_3 (nm)	d_4 (nm)	$\alpha_{\rm gr}$ (%)	α_0 (%)
A	28	0.33	56	2	0.62	2.3
B	51	0.33	36	1.33	0.61	2.3

Supplementary Table 2. **Thickness parameters and calculated absorbance values of GJJ devices.** Absorbance calculations are performed assuming 1550 nm photons that are normally incident on the GJJ devices.

Now we calculate the reflectivity of the heterostructure, $\mathcal{R}_{\rm hs}$. The flow of optical power is determined by Poynting vectors, S_i^β:

$$S_i^\beta = {\rm Re}(E_i^\beta H_i^{*\beta}) \quad (7)$$

where H_i^β is the magnetic field amplitude of light traveling in the i-th optical layer in the β direction. The electric and magnetic fields are related by $H_i^\beta = n_i E_i^\beta / Z_0$, with $Z_0 = \sqrt{\mu_0/\epsilon_0}$ being the free-space impedance, ϵ_0 the vacuum permittivity, and μ_0 the vacuum permeability. Hence, S_0^+, S_0^- and $S^+_{\rm sub}$ are given by:

$$S_0^+ = \frac{|E_0^+|^2}{Z_0} \quad (8)$$

$$S_0^- = \left|-\frac{m_{21}}{m_{22}}\right|^2 S_0^+ \quad (9)$$

$$S^+_{\rm sub} = n_{\rm sub}\left|m_{11} - \frac{m_{12}m_{21}}{m_{22}}\right|^2 S_0^+ \quad (10)$$

We can calculate $\mathcal{R}_{\rm hs}$ using:

$$\mathcal{R}_{\rm hs} = \frac{S_0^-}{S_0^+} = \left|-\frac{m_{21}}{m_{22}}\right|^2 \quad (11)$$

and the absorption coefficient, α_l, of the l-th layer using [19, 21]:

$$\alpha_l = \frac{(S_0^+ - S_0^-) - (S_l^+ - S_l^-)}{S_0^+} \quad (12)$$

Using the partial wave-transfer matrix, X:

$$\begin{pmatrix} E^+_{\rm sub} \\ 0 \end{pmatrix} = X \begin{pmatrix} E^+_l \\ E^-_l \end{pmatrix} \quad (13)$$

where

$$X = \begin{cases} M_{\rm sub,4}M_4\left(\prod_{i=l}^{3} M_{i+1,i}M_i\right) & 1 \le l \le 3 \\ M_{\rm sub,4}M_4 & l = 4 \end{cases} \quad (14)$$

$$= \begin{pmatrix} x_{11} & x_{12} \\ x_{21} & x_{22} \end{pmatrix} \quad (15)$$

we have:

$$S_l^+ = n_l\left|\frac{m_{11} - m_{12}m_{21}/m_{22}}{x_{11} - x_{12}x_{21}/x_{22}}\right|^2 S_0^+ \quad (16)$$

$$S_l^- = n_l\left|\frac{m_{11} - m_{12}m_{21}/m_{22}}{x_{12} - x_{11}x_{22}/x_{21}}\right|^2 S_0^+ \quad (17)$$

Material	$\mathcal{R}$
Si	0.31
Device A	0.21
Device B	0.21

Supplementary Table 3. **Calculated $\mathcal{R}$ values of 1550 nm photons at normal incidence from vacuum.** $\mathcal{R}$ of the relevant materials on the graphene SPB chip at a photon wavelength of 1550 nm. We calculate the $\mathcal{R}$ of Si from the Fresnel equations, $\mathcal{R} = |(1-n)/(1+n)|^2$, where n is the refractive index of Si. We calculate $\mathcal{R}$ of the graphene SPBs using Eqn. 11 from the wave-transfer matrix method. We experimentally determine $\mathcal{R}$ of MoRe from reflectometry measurements to be ~ 0.85.

$$\alpha_l = 1 - \left|\frac{m_{21}}{m_{22}}\right|^2 - n_l\left(\left|\frac{m_{11}-m_{12}m_{21}/m_{22}}{x_{11}-x_{12}x_{21}/x_{22}}\right|^2 - \left|\frac{m_{11}-m_{12}m_{21}/m_{22}}{x_{12}-x_{11}x_{22}/x_{21}}\right|^2\right) \tag{18}$$

Using the measured thickness listed in Table 2, we find that $\alpha_{\mathrm{gr}} \approx 0.6\%$, lower than the free-standing value, α_0, of 2.3%. Although our experimental setup would not allow us to directly measure α_{gr}, we find that the wave-transfer matrix method provides an excellent agreement on the calculated ratio of the reflectance values of the graphene heterostructure to silicon (Table 3) with the measured value (gray dashed lines, Figure 2A).

Supplementary Note 4: Scanning laser reflectometry and estimation of the beam spot size

We use the laser reflectometry setup (Supplementary Figure 5) described in the Methods section to locate our beam spot with respect to our device. Supplementary Figure 6 compares an optical image of Device A with our scanned reflectometry image taken at 140 mK. The highly reflective superconducting MoRe electrodes and gate leads are clearly discernible as bright yellow regions in Supplementary Figure 6B. Below the junction, the graphene heterostructure appears as the darker red region. These features enable us to precisely place the beam spot on our graphene SPB.

Experimentally, we determine the beam spot size, $2w_o$, from V_{refl} in Supplementary Figure 6B and Supplementary Figure 8A. $V_{\mathrm{refl}}(x,y)$ images the graphene heterostructure with a point spread function given by the Gaussian beam profile of the beam spot, $I(x,y)$, through a single-mode optical fiber such that:

$$V_{\mathrm{refl}}(x,y) \propto \iint \mathcal{R}(x',y') I(x-x', y-y') \mathrm{d}x'\mathrm{d}y' \tag{19}$$

where $\mathcal{R}$ is the reflectance, and

$$I(x,y) = I_0 \exp\left(\frac{-8(x^2+y^2)}{(2w_o)^2}\right) \tag{20}$$

with $I_0 = 8P_{\mathrm{laser}}/\pi(2w_o)^2$ being the intensity at the center of the beam spot, and P_{laser} being the laser power through the optical fiber. Using the values of $\mathcal{R}$ for hBN-encapsulated graphene and silicon calculated in Table 3, we fit the V_{refl} data in Figure 2A and Supplementary Figure 8A with the convolution integral (Eqn. 19) for the beam spot size. The best fitted values of $2w_o$ are given in Table 4.

This beam spot size, however, is larger than the diffraction limit, $2w_o^{\mathrm{dl}}$, given by:

$$2w_o^{\mathrm{dl}} = \frac{\lambda}{2\mathrm{NA}} \tag{21}$$

where NA is the numerical aperture of the focusing lens. We can reconcile the discrepancy by considering the underfilling of light through the focusing lens[23, 24]. We calculate the enlarged beam spot size[24], $2w_o'$:

$$2w_o' = K\lambda f_{\#} \tag{22}$$

where $f_{\#}$ is the f-number, defined as the ratio between the focal length and diameter, ϕ_{lens}, of the focusing lens. The beam spot constant, K, is given by[24]:

$$K = 1.654 - \frac{0.105}{\tilde{\phi}} + \frac{0.28}{\tilde{\phi}^2} \tag{23}$$

where $\tilde{\phi} \equiv \phi_{\mathrm{coll}}/\phi_{\mathrm{lens}}$ is the truncation ratio, with ϕ_{coll} being the diameter of collimated light entering the focusing lens. For both GJJ SPB devices, $\phi_{\mathrm{coll}} = 3.6$ mm. The agreement between the extracted beam spot size and our calculation of $2w_o'$ for both devices suggests that the underfilling of the lens causes the beam spot size in our experiment larger than the diffraction limit.

Supplementary Note 5: Estimating the quantum efficiency

Experimentally, we infer η in Figure 1D, 2C and 2F by $\eta = \Gamma_{\mathrm{meas}}/\dot{\mathcal{N}}_{\mathrm{abs}}$, where $\dot{\mathcal{N}}_{\mathrm{abs}}$ is the photon rate absorbed by the graphene. $\dot{\mathcal{N}}_{\mathrm{abs}}$ is the product of the incident photon rate $\dot{\mathcal{N}}$ and α_{gr}, i.e. $\dot{\mathcal{N}}_{\mathrm{abs}} = \alpha_{\mathrm{gr}}\dot{\mathcal{N}}$. The incident photon rate is determined by both the spatial overlap of the laser spot with the graphene, and the laser power that is applied on the device. As a function of position, $\dot{\mathcal{N}}(x,y)$

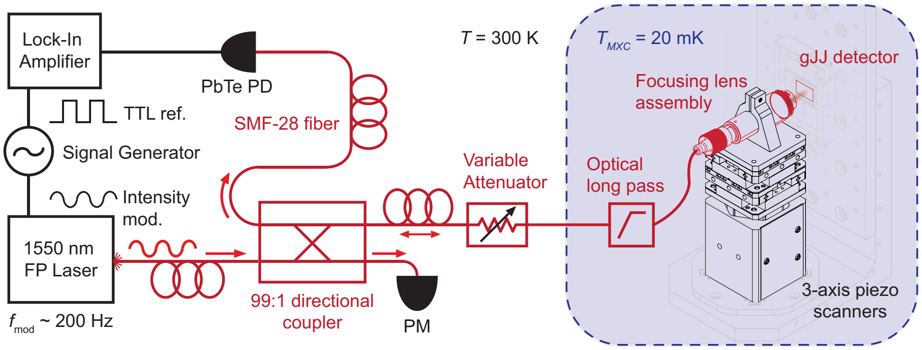

Supplementary Figure 5. **Reflectometry schematic.** Schematic of the reflectometry setup used to locate and position our laser spot onto the graphene. Laser light from an intensity-modulated 1550 nm Fabry-Perot (FP) laser is routed through an SMF-28 single-mode fiber onto the graphene SPB. This light is then reflected off the detector and routed to a PbTe photodetector (PD) that transduces an AC-modulated electrical signal proportional to the reflected light intensity hitting the PbTe PD. The AC-modulated electrical signal is then demodulated and amplified using a lock-in amplifier. The RMS power of our laser light as measured by an optical power meter (PM) is $\sim$200 μW. Upon the 20 dB attenuation passing through the directional coupler and roughly 3 dB of loss from insertion loss between fiber components and the focusing lens assembly, we estimate that 1 μW RMS of laser power is incident on the graphene SPB during the reflectometry measurements.

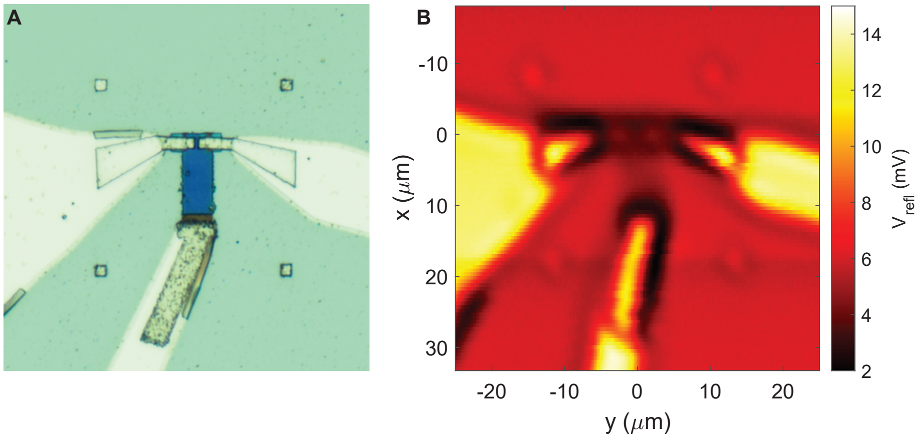

Supplementary Figure 6. **Reflectometry measurement used to find the graphene target. (A)** Optical image of Device A. **(B)** 2D reflectometry scan of Device A taken at 140 mK.

Device	A	B
NA	0.3	0.6
$f_{\#}$	1.7	0.8
$\phi_{\rm lens}$ (mm)	5.5	6.5
$\phi_{\rm coll}$ (mm)	3.6	3.6
$\tilde{\phi}$	0.65	0.55
K	2.2	2.4
diffraction-limited $2w_o^{\rm dl}$ (μm)	2.6	1.3
finite-size $2w_o'$ (μm)	5.7	3.1
fitted $2w_o$ (μm)	7	4

Supplementary Table 4. **Optical system parameters and beam spot sizes.** Collimating and focusing lens parameters used in the calculation of the enlarged beam spot size, $2w_o'$, for both GJJ SPB devices.

is calculated by performing a convolution between the laser intensity, $I(x,y)$, with the graphene binary profile, $\mathcal{G}(x,y)$, normalized to the energy of a single photon, $h\nu$:

$$\dot{\mathcal{N}}(x,y) = \frac{1}{h\nu}\iint \mathcal{G}(x',y')I(x-x',y-y')\mathrm{d}x'\mathrm{d}y' \quad (24)$$

where $\mathcal{G}(x,y) = 1$ on the graphene heterostructure, and 0, otherwise. When the beam spot is centered on the middle of the graphene heterostructure, with $P_{\rm laser} = 1$ fW, $\dot{\mathcal{N}} \simeq 7.3$ kHz (Device B) and $\dot{\mathcal{N}}_{\rm abs} \simeq 45$ Hz based on the value of $\alpha_{\rm gr}$ in Table 2. In the I_b ranges where $\Gamma_{\rm meas}$ plateaus at about 35 ± 4 Hz in Figure 1D, $\eta \simeq 0.78 \pm 0.08$.

Supplementary Note 6: Estimation of the Impact of a Plasmon Mode

Previous work on GJJ SPDs relied on the presence of a plasmon mode at the NbN-graphene interface to enhance

Reference	$\mathcal{A}_{\text{eff}}$ (μm^2)	$\langle\alpha\rangle$	$\dot{\mathcal{N}}_{\text{abs}}$ (Hz)
Plasmon	$2 \times 2.8 \times 0.19$	0.6	371
Graphene	4π	6.1×10^{-3}	45

Supplementary Table 5. **Comparison of single-photon absorption rate between the plasmon mode and graphene.** Relevant parameters for the calculation of $\dot{\mathcal{N}}_{\text{abs}}$. In the calculations of $\dot{\mathcal{N}}_{\text{abs}}$ from both the GJJ and the plasmon mode, $\mathcal{J}_{\text{photon}} = 581$ photons per second per μm^2 per 1 fW, and $P_{\text{laser}} = 1$ fW.

the photon absorption efficiency [25]. While the detection mechanism in the previous experiment is through Cooper pair breaking rather than the thermal effect in this report, here we consider how the presence of a plasmon mode, if it exists, would impact Γ_{meas}. To begin, we calculate the number of incident photons per unit time per unit area per unit incident laser power through the focusing lens, $\mathcal{J}_{\text{photon}}$, given by:

$$\mathcal{J}_{\text{photon}} = \dot{\mathcal{N}}/\pi w_o^2 \tag{25}$$

with $\dot{\mathcal{N}}$ being the photon rate in the Gaussian beam and πw_o^2 the beam spot area. We find $\mathcal{J}_{\text{photon}} = 581$ photons per second per μm^2 per 1 fW of laser power.

If a similar plasmonic mode exists, based on Ref. [26], we can estimate an effective single-photon absorption area, $\mathcal{A}_{\text{eff}}$, and averaged photon absorption coefficient, $\langle\alpha\rangle_{\text{plasmon}}$, to calculate the expected absorbed photon rate due to a plasmon, $\dot{\mathcal{N}}_{\text{abs}}^{(\text{plasmon})}$:

$$\dot{\mathcal{N}}_{\text{abs}}^{(\text{plasmon})} = \mathcal{J}_{\text{photon}}\mathcal{A}_{\text{eff}}\langle\alpha\rangle_{\text{plasmon}}P_{\text{laser}} \tag{26}$$

We find that $\dot{\mathcal{N}}_{\text{abs}}^{(\text{plasmon})}$ would be 371 Hz. This value is ∼8 times more efficient than direct absorption by the graphene layer (Table 5), which would result in a sizable enhancement in Γ_{meas} when the beam spot is centered on the GJJ. Instead, we observe a reduced Γ_{meas} in Figure 2, suggesting that the plasmon mode is either substantially less effective than in Ref. [26] or not present in the graphene-MoRe interface. We attribute the difference to the graphene-superconductor interface — the superconducting electrodes are made of MoRe in this report rather than NbN in the previous one. Regardless, the data suggests that plasmonics play little to no role in our experiment.

Supplementary Note 7: Single-Photon Detection in a Second Device

(Single-photon sensitivity.) In addition to the device which was studied in the main text, we have also confirmed single-photon detection in a second device (Device A). Parameters for both devices are listed in Table 1. We confirm the single-photon detection in the same manner as the main text by placing the beam spot 5 μm from the junction and applying a fixed I_b at ∼78% of I_c while measuring Γ_{meas}. As with Device B, we find that the switching events adhere to Poissonian statistics (Supplementary Figure 7A inset), indicating the detector is shot-noise limited. We plot the switching probability against P_{laser} and find a linear trend (Supplementary Figure 7A), indicating that the switching events are due to the detection of a single photon.

(Gate dependence.) In order to compare the two devices, we explore the performance of Device A on electron density. As V_{gate} increases from the charge neutrality point (≃0.0 V), Γ_{meas} becomes considerably larger than Γ_{dark} (Supplementary Figure 7B), similar to the data from Device B shown in Figure 3. The nonlinear Γ_{meas} in the log-linear plot in Supplementary Figure 7B indicates that Γ_{meas} is not proportional to $\sim \exp -\Delta U$, i.e. in activation theory. Comparison of the experimental data and theory [25, 27] shows that this switching behavior of a Josephson junction is due to discrete triggering event, i.e. single photons, rather than the induction from a thermal bath in equilibrium. This is an important distinction of our graphene SPB [28] from graphene bolometers [18, 27, 29–41].

Up to the highest attainable V_{gate} in Device A (the bottom hBN layer is 20 nm thicker than Device B), Γ_{meas} does not fully saturate. This is despite the n_e explored for Device A falling within the range of n_e that provided robust bias saturation in Device B. Moreover, η for Device A is considerably less than that of Device B. The correlation between of the non-saturating $\Gamma_{\text{meas}}(I_b)$ and a lower η is consistent with previous studies [25, 42].

Similar to Figure 3D and E for Device B, we study the thermal effect of and η-vs.-Γ_{dark} tradespace of Device A. Supplementary Figure 7C plots η vs. $\Delta U/k_B$ at several V_{gate}. At the highest gate voltage, $V_{\text{gate}} = 6$ V, a single photon can induce phase-particle escape from a $\Delta U/k_B \sim 2$ K with $\eta \sim 0.5$. We also plot η vs Γ_{dark} in Supplementary Figure 7D and find that at the same gate voltage, Device A can detect a single photon with $\eta \sim 0.5$ for Γ_{dark} at 1 Hz. These figures of merit are lower than that of Device B.

(Spatial scanning.) To further explore this difference in single-photon detection between the devices, we study the spatial dependence of Device A. As in the main text, we position the center of our beam spot ∼5 μm below the edge of the superconducting contact and scan the laser along the y-direction of the graphene heterostructure. The incident laser power is fixed at 200 aW. As we traverse the graphene target, the reflectance signal decreases (8A) and Γ_{meas} increases (Supplementary Figure 8B) as the beam spot moves towards the center of the graphene. When the spot is centered on the graphene, Γ_{meas} reaches a maximum and plummets when the beam spot scans off the opposite edge of the graphene. We calculate $\dot{\mathcal{N}}_{\text{abs}}$ using the convolution integral (Eqn. 24) of $\dot{\mathcal{N}}$ and α_{gr} in Table 2 and retrieve η as a function of position (Supplementary Figure 8C).

We now turn to scanning the laser across the longitudinal extent of the device. The beam is centered along

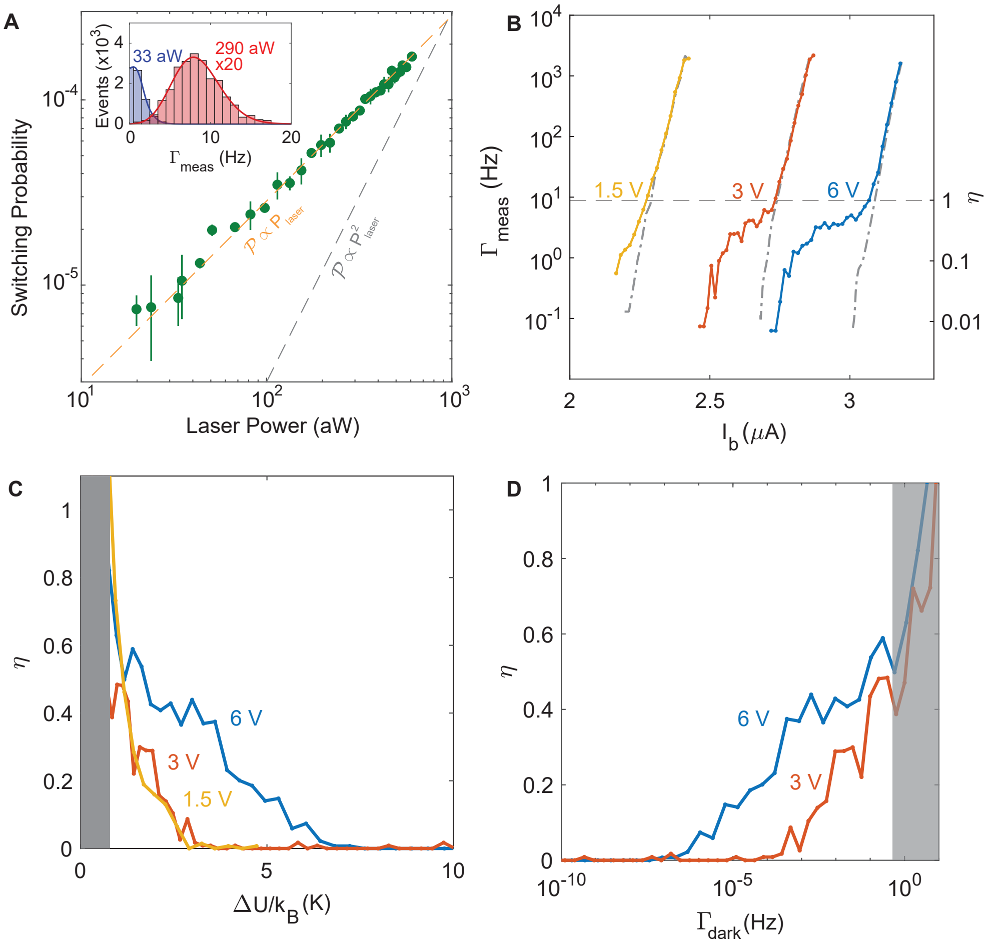

Supplementary Figure 7. **Single-photon signal in Device A. (A)** Switching probability as a function of applied laser power. Linearity indicates that the switching events are caused by a single photon. Error bars are the standard deviation over multiple runs. Inset: The switching events adhere to Poissonian statistics indicating that the device is shot-noise limited. **(B)** $\Gamma_{\rm meas}(V_{\rm gate})$ for Device A with $P_{\rm laser}$ = 200 aW. **(C)** η vs. $\Delta U/k_B$ shows that at the highest $V_{\rm gate}$ = 6 V, a single photon can induce the escape of the GJJ phase particle from a $\Delta U/k_B$ of $\sim$ 2 K with $\eta = 0.5$. Gray box indicates the region where self-switching of the junction is dominant. **(D)** Tradespace between η vs $\Gamma_{\rm dark}$ shows Device A can detect a single photon with $\eta \sim 0.5$ for $\Gamma_{\rm dark}$ at 1 Hz. Gray box indicates the region where self-switching of the junction is dominant.

the horizontal extent of the graphene target and scanned along the x-direction of 7. We measure the reflectance signal (Supplementary Figure 8D), along with $\Gamma_{\rm meas}$ as a function position away from the GJJ (denoted by shaded yellow region in Supplementary Figure 8D-F). Again, we find that geometric effects dominate: $\Gamma_{\rm meas}$ is predominantly proportional to the geometric overlap between the beam and graphene target. It is important to note that Device A is roughly half the longitudinal length of Device B. Therefore, we do not see the flat plateau in $\Gamma_{\rm meas}$ observed in Figure 2.

Supplementary Figure 8F shows η in the longitudinal direction after accounting for the overlap of the beam spot with the graphene heterostructure (Eqn. 24). When the beam spot is positioned over the leads we observe a depression in η. Notably, we find that along the longitudinal extent of Device A, η never reaches the η observed in Device B. While the exact reason for this observation is beyond the scope of this manuscript, we note that the operation n_e, $\langle I_s \rangle$ and I_r between Devices A and B are roughly the same. Therefore, we speculate that the device design is the primary reason for the comparatively smaller η in Device A. Notably, Device A uses 1D contacts, while Device B uses 2D contacts. It is possible that the different fabrication methods may result in different densities of resonant scatterers[43] at the graphene-superconductor interface, which could increase the cooling rate of the graphene electrons. Experiments using different fabrication methods and different sizes of graphene absorber will need to optimize the SPD performance.

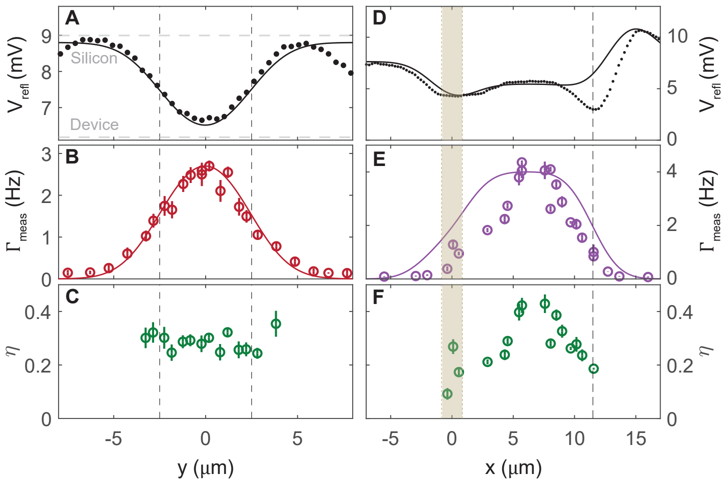

Supplementary Figure 8. **Scanning the photon input position across the graphene (A-C)** in the transverse (y-) direction. Vertical dashed lines marks the graphene location. **(D-F)** Scanning in longitudinal (x-) direction. Vertical purple region designates the Josephson-junction location. Vertical, gray dashed line marks the graphene location. **(A, D)** The transverse reflectance signal (dots) and fitted step response based on the dielectric constants of graphene and silicon, interference effects, and geometric convolution assuming a Gaussian beam of spot size, 7 μm (solid line). The decrease in reflectance signal in **(A)** at the extremities of the transverse scan result from the beam spot clipping the edge of the nearby MoRe electrodes. **(B, E)** The measured switching rate (open circles) and expected switching rate based off of the step response (solid line). **(C, F)** The extracted η (open circles) when the beam is over the device. Error bars are the standard deviation over multiple runs.

Supplementary Note 8: Extended data on the Poisson Statistics

As mentioned in the main text, the time-binned switching events measured at various laser powers adhere to Poissonian statistics. This indicates that our devices are shot-noise limited. In Supplementary Figure 9, we show the extended distributions for several laser powers for both devices. The solid lines are the Poissonian fit.

Supplementary Note 9: Single-photon detection at 1.2 K

In order to verify SPD at elevated temperatures, we repeat the procedure described in the main text. At $T_0 = 1.2$ K, we again apply a fixed I_b to the junction at roughly 90% of I_c and measure Γ_{meas}. As with low temperature, we find that Γ_{meas} adheres to Poissonian statistics (Supplementary Figure 10 inset), indicating the detector is still shot-noise limited at 1.2 K. We plot Γ_{meas} against P_{laser} and find a linear trend (Supplementary Figure 10), indicating that the switching events are due to the detection of a single photon. Therefore, our device operates as an SPD up to 1.2 K.

Supplementary Note 10: Macroscopic Quantum Tunneling Fits of the Junction

To show that in the absence of illumination our device is nominally operated in the MQT regime, we fit Γ_{meas} as a function of I_b to the MQT model. Specifically, our switching rate is described as $\Gamma_{\text{meas}} = A\exp(-\Delta U/k_B T_{\text{eff}})$, where, in the MQT regime [44]:

$$A = A_{\text{MQT}} = 12\omega_p\sqrt{\frac{3\Delta U}{2\pi\hbar\omega_p}} \tag{27}$$

and

$$T_{\text{eff}} = \hbar\omega_p/[7.2k_B(1+0.87/Q)] \tag{28}$$

where $\omega_p = \omega_{p0}(1-\gamma_{\text{JJ}}^2)^{\frac{1}{4}}$ is the junction plasma frequency, $\omega_{p0} = (2eI_c/\hbar C_{\text{JJ}})^{\frac{1}{2}}$ is the zero bias plasma frequency, $C_{\text{JJ}} = \hbar/R_n E_{\text{Th}}$ is the junction shunting capacitance [6], with R_n as the normal state resistance, and $E_{\text{Th}} = \hbar\mathcal{D}/L^2$ as the Thouless energy, where L is the channel distance of GJJ, $\mathcal{D} = v_F l_{\text{mfp}}/2$ is the diffusion constant, with l_{mfp} as the mean free path, $\gamma_{\text{JJ}} = I_b/I_c$ is the normalized bias current, $Q = \omega_p R_n C_{\text{JJ}}$ is the junction quality factor, $\Delta U = 2E_{\text{J0}}(\sqrt{1-\gamma_{\text{JJ}}^2} - \gamma_{\text{JJ}}\cos^{-1}\gamma_{\text{JJ}})$ is the phase particle barrier height and $E_{\text{J0}} = \hbar I_c/2e$ is the Josephson energy. Here, e is the electron charge.

In Supplementary Figure 11, we show a fit for each of the V_{gate} displayed in Figure 3A. We find good agreement between the data and fits. Notably, at higher V_{gate},

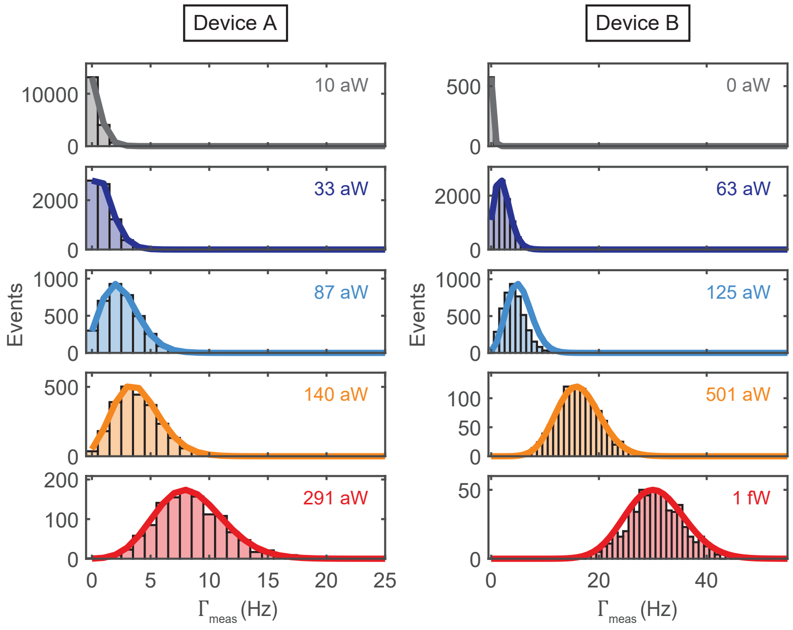

Supplementary Figure 9. **Extended Poissonian statistics for both devices.** The distributions of time-binned switching events with several applied laser powers adhere to Poissonian statistics, indicating that our devices are shot-noise limited.

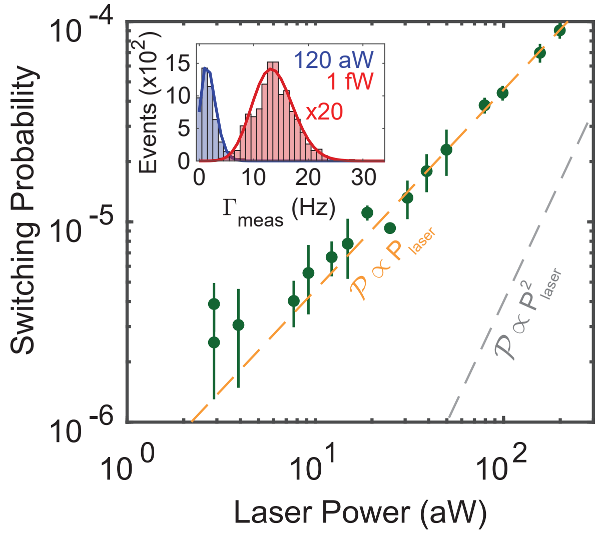

Supplementary Figure 10. **Single photon signal at 1.2 K.** Linearity of the switching probability as a function of laser power. **Inset** Poisson distributions show the device is still shot-noise limited at 1.2 K. Error bars are the standard deviation over multiple runs.

the fitted value for resistance begins to increase with increasing V_{gate}. This trend is not observed in the lock-in measurements of R_n. This trend of increasing resistance is further accompanied by an increase in I_c and a qualitative bend in Γ_{meas} versus I_b. This may be due to the device matching to a resonance in the electrode wiring, which in turn produces a non-thermal noise as was observed in Ref. [45]. This noise likely inhibits SPD at higher densities.

In addition to the MQT regime, at higher temperatures the device can enter the thermally activated (TA) regime whereby the thermal fluctuations cause the phase particle to excite over ΔU. In the TA regime, $T_{\text{eff}} = T_e$ and [44]:

$$A = A_{\text{TA}} = \frac{\omega_p}{2\pi}\left(\sqrt{1+\frac{1}{4Q^2}} - \frac{1}{2Q}\right) \tag{29}$$

Supplementary Note 11: Calculation of the NEP

In Supplementary Figure 12, we show the η, Γ_{dark} and NEP of from Figure 1d. As stated in the main text, we calculate NEP as $\epsilon_P\sqrt{\Gamma_{\text{dark}}}/\eta$. We find, for a Γ_{dark} of 1 photon/week, we have an $\eta \sim 0.75$, corresponding to an NEP of 2×10^{-22} W/$\sqrt{\text{Hz}}$.

Supplementary Note 12: Simple modeling of detection efficiency versus temperature and ΔU

Figure 4 shows that the calculated $\eta(T_0, \Delta U/k_B)$ qualitatively agrees with the experimental data. We calculated η using [28]:

$$\eta = 1 - \exp\left(-\int \Gamma_{\text{total}}(t)\right)dt) \tag{30}$$

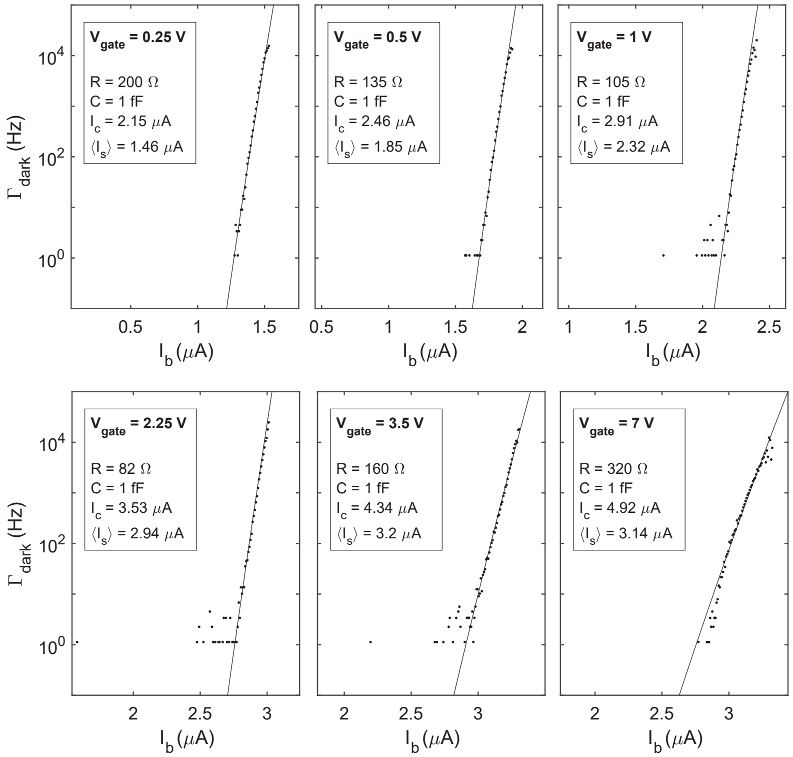

Supplementary Figure 11. **MQT Fits for the Data Presented in the Main Text.** We use the Fulton-Dunkleberger technique [45] to extract the dark count rate, $\Gamma_{\rm dark}$, for several representative gate voltages, $V_{\rm gate}$. We then fit using the expressions describing MQT by varying the resistance (R) and critical current, I_c for each $V_{\rm gate}$. Junction capacitance, C, is held constant.

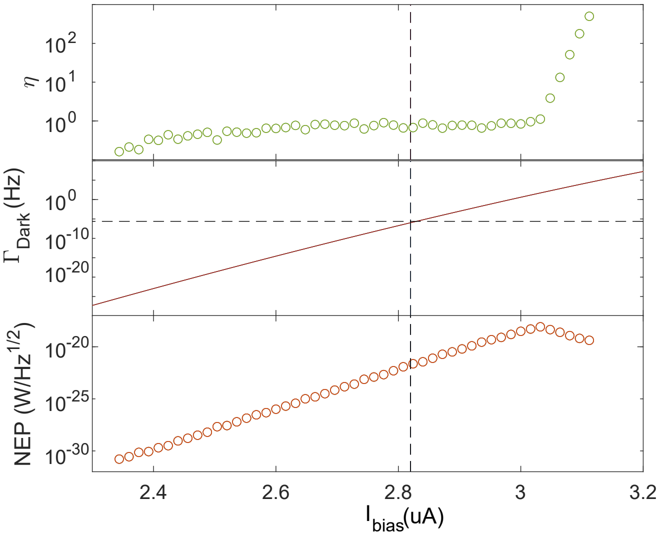

Supplementary Figure 12. **Noise Equivalent Power Calculation** Top: η plotted against $\mathrm{I_{bias}}$ reproduced from Figure 1D. Vertical black dashed line corresponds to $\eta \sim 0.75$ and $\Gamma_{\rm dark} = 1$ photon/week Middle: $\Gamma_{\rm dark}$ extracted from the MQT fit from Figure 1dD Bottom: Noise equivalent power of the data presented in Figure 1D. At an $\mathrm{I_{bias}}$ of 2.82 μA, corresponding to an $\eta \sim 0.75$ and a $\Gamma_{\rm dark}$ of 1 photon/week, we find an NEP of $\sim 2 \times 10^{-22}$ W/$\sqrt{\rm Hz}$

where $\Gamma_{\rm total}$ is the total switching rate of the GJJ, i.e. $\Gamma_{\rm total} = \Gamma_{\rm MQT} + \Gamma_{\rm TA}$. To induce the phase particle out of the washboard potential, i.e. switching of the GJJ, we can expect the switching rate to follow the general form, $\Gamma = A \exp\left(-\Delta U / k_B T_{\rm eff}\right)$ based on activation theory. In our devices, we nominally operate the GJJ in the MQT regime such that $\Gamma_{\rm MQT}$ is dominant. However, when the graphene absorbs a photon, the rise in T_e will increase $\Gamma_{\rm TA}$, causing a TA switching event. Therefore, achieving a high η over the dark count rate requires that the photon to elevate $\Gamma_{\rm TA} \gg \Gamma_{\rm MQT}$. In this case, we can approximate that $T_e = T_{1p}$ so that $\Gamma_{\rm total} \simeq \Gamma_{1p} = A \exp\left(-\Delta U / k_B T_{1p}\right)$.

Increasing T_0 can degrade SPB in two ways. First, increasing ambient thermal fluctuations raises the dark $\Gamma_{\rm TA}$ and reduces I_c of the GJJ. However, the $\langle I_s \rangle$ changes only by $\sim 30\%$ over the temperature range studied (a similar change in $\langle I_s \rangle$ is observed when reducing $V_{\rm gate}$ from 2 V to 1 V). We shall neglect the change of the GJJ's ability to detect a single photon in our model, i.e. the T_0 dependence of A and ΔU, as temperature rises.

The second effect, the thermal response of the graphene electrons, can dominate the suppression of η when T_0 rises. Since $T_{1p} = \sqrt{2h\nu/\gamma_s \mathcal{A} + T_0^2}$ [28] with $\mathcal{A}$ being the total area of the monolayer graphene when a uniform T_e is reached [46], T_{1p} changes considerably when the two terms inside the square root become com-

parable. Moreover, the integration time in Eqn. 30 is limited by τ_{ep}. Since $\tau_{\mathrm{ep}}(T_0) \propto \tau_{\mathrm{ep}}(T_0 = 20\ \mathrm{mK})T_0^{2-\delta}$, it reduces quickly as T_0 rises. Owing to the relatively large area-to-perimeter ratio of the graphene used in our experiment, it is likely that the E-Ph coupling is in the clean limit ($\delta = 4$) [30, 47–52] rather than the disorder or resonant-scattering limit ($\delta = 3$) [43, 53–57].

To calculate η, we simplify Eqn. 30 by assuming that the device stays at Γ_{1p} for τ_{ep}:

$$\eta = 1 - \exp(-\Gamma_{1p}(T_0)\tau_{\mathrm{ep}}(T_0)) \qquad (31)$$

where we set A in Γ_{1p} such that $A = A_{\mathrm{TA}}(T_0 = 20\ \mathrm{mK})$.

Using Eqn. 31, we vary ΔU and T_0 to calculate η (Figure 4B). We find qualitative agreement between the experiment and calculation when $\tau_{\mathrm{ep}}(T_0 = 20\mathrm{mK})$ is 75 ns and $T_{1p}(T_0 = 0)$ is 2.5 K. We note, this model does not capture the behavior at high $\Delta U/k_B$ as it does not account for the retrapping of the junction. However, we see that the overall reduction in η for rising T_0 is consistent with the experiment. This indicates that our assumption about the unchanging ability of the GJJ to detect a single-photon in this temperature range is reasonable and that the dominant reduction in η is due to thermal effects of the graphene electrons.

Supplementary Note 13: Calculation of the characteristic length scale of heat diffusion

The characteristic length scale of heat diffusion determines the reduction of η at a distance away from the Josephson junction. In the main text we estimate that this length scale to be $\sim$230 μm. For this we take electrical conductivity $\sigma = 0.02$ S (inferred from the resistance measurement listed in Table 1) and $\gamma_s = 6.71$ $\mathrm{Ws^{-2}K^{-2}}$ (corresponding to $n_e = 10^{12}\ \mathrm{cm^{-2}}$). Therefore, $\mathcal{D} = \sigma\mathcal{L}_0/\gamma_s = 0.727\ \mathrm{m^2/s}$. Taking a characteristic E-Ph interaction time found in Figure 4 of $\tau_{\mathrm{ep}} = 75$ ns, we find $l_D = \sqrt{\mathcal{D}\tau_{\mathrm{ep}}} \simeq 230\ \mu\mathrm{m}$. As stated in the main text, this length scale is much larger than the device's longitudinal length and likely explained the constant η over the device.

Supplementary Note 14: Microscopic understanding of the switching mechanism

In superconducting-normal-superconducting Josephson junctions, supercurrent is mediated via Andreev reflection. Here, an incoming electron retroreflects off of a superconducting electrode as a hole (and vice versa). In a Josephson junction, repeated Andreev reflections between electrons and holes generate Andreev bound states – an energy spectrum which is dependent on the weak-link material, superconducting gap and phase.

The supercurrent mediated by the Andreev spectrum is $\Sigma_i dE_i/d\varphi_i f(E)$ i.e. the sum of the slopes of the occupied Andreev bands. At low temperatures, the Andreev spectrum allows for the maximum measured supercurrent through the device, however as temperature rises, the electron-hole symmetry of the spectrum allows for states with opposite current contributions to become occupied, thus lowering the critical current mediated by the junction.

In the case of the graphene SPD, an incoming photon is absorbed by the graphene electrons, causing an increase in temperature that propagates towards the junction (see [46]), while the superconducting leads remain at the bath temperature. The leads are largely unaffected due to their sizable superconducting gap and strong thermal anchoring, whereas the hot electrons in graphene broaden the distribution that feeds the ABSs. This temperature rise increases the occupation of states above the Fermi level, thereby reducing the critical current. Fundamentally, the Andreev spectrum enables this work in two ways: 1) The Andreev spectrum necessitates that the critical current is dependent on the graphene electronic temperature, enabling the mapping from the microscopic of the junction to the macroscopic circuit parameters (i.e. the change in critical current)" 2) The semi-continuous Andreev spectrum reduces the energy scaling from the superconducting gap, to the lower energy scale of the Andreev level spacing. In the limit of many modes this is nearly continuous and allows us to unlock the zero-bandgap of the graphene electrons.

Supplementary Note 15: Behavior of η as a function of position from the JJ detector

The persistence of a high quantum efficiency η even when the beam spot is far away from GJJ can be understood through simple modeling of electron heat diffusion in graphene. In our device, electronic heat simultaneously diffuses out through electron-electron interactions and dissipates via collision with the lattice [58], as modeled by Eqn. 1. Following Ref. [28, 46, 59], the value of τ_{ep} for different devices can be estimated by $\tau_{\mathrm{ep}} = \gamma_S/\delta\Sigma T_0^{\delta-2}$. The power law δ and the strength of E-Ph coupling Σ take on different values depending on the mechanism of E-Ph scattering. In the limit of l_{mfp} larger than the typical inverse phonon momentum, heat dissipation follows a $\delta = 4$ power law, and $\Sigma = \pi^{5/2}k_B^4 D^2 n_e^{1/2}/(15\rho_m\hbar^4 v_F^2 s^3)$ [47, 52] where $D \simeq$ 18 eV is the deformation potential, $\rho_m = 7.4 \times 10^{-19}$ kg $\mu\mathrm{m}^{-2}$ is the mass density of graphene, and $s = 2.6 \times 10^4\ \mathrm{m\ s^{-1}}$ is the speed of sound in graphene [60]. If l_{mfp} is lower, heat dissipation is governed by defect-assisted scattering, and follows a $\delta = 3$ power law, with $\Sigma = 2\zeta(3)k_B^3 D^2 n_e^{1/2}/(\pi^{3/2}\rho_m\hbar^3 v_F^2 s^2 l_{\mathrm{mfp}})$ [53, 55]. Experimentally, however, a third regime where E-Ph coupling is dominated by resonant scattering on the edge of the graphene is often observed [27, 43, 61], especially for samples with a high edge-to-surface ratio. In this

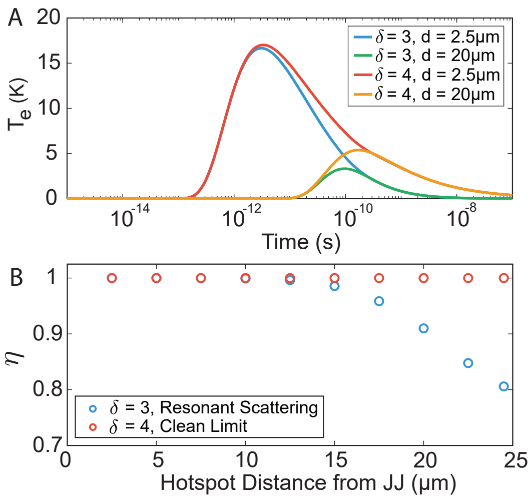

Supplementary Figure 13. **Numerical simulations of thermal propagation and thermal switching.** Simulations calculated numerically for a graphene SPB 25 μm in length. Panel A shows the time dependent electron temperature measured at different distances from the JJ. Panel B shows the detection efficiency η as a function of the hotspot's distance from the Junction. Charge carrier density is set at 2.0×10^{12} cm^{-2}, and numerical values of the E-Ph coupling constant Σ are 0.031 Wm^{-2}K^{-4} and 1 Wm^{-2}K^{-3} for $\delta = 4$ and $\delta = 3$, respectively.

regime, heat dissipation also follows a $\delta = 3$ power law, but has a higher value of Σ, with experimental values close to 1 Wm^{-2}K^{-3} [27, 56, 61]. While it is difficult to determine with certainty what E-Ph coupling regime our device is in (especially when accounting for the possibility of local differences in scattering across the length of the device), modeling the thermal behavior of a device with our graphene SPB's dimensions and parameters in both the diffusive ($\delta = 4$ clean limit) and dissipative ($\delta = 3$ resonant scattering) cases can help us understand the range of possibilities we can expect to see for the distance dependence of η.

First, we determine our initial condition for solving Eqn. 1. The absorption of a single near-infrared photon by graphene causes an inter-band electronic excitation, which then results in an initial cascade of hot electron-electron interactions [58, 62, 63]. We model this initial heating as a Gaussian hotspot with half-width-half-maximum ξ [64, 65]:

$$T_e(t=0) = T_{\text{hot}} e^{-(x-x_0)^2/\xi^2} + T_0 \tag{32}$$

where T_{hot} is the average temperature of the hot electrons in the initial photo-excitation cascade, and x_0 is the distance of the hotspot from the center of the graphene. The relationship between the hotspot size ξ and hotspot temperature T_{hot} can be found through integrating electronic heat capacity $C_e = \mathcal{A}\gamma_S T_e$ with respect to temperature [28, 30, 31, 59]:

$$\int dE = \int_{T_0}^{T_{\text{hot}}} \mathcal{A}\gamma_S T_e \, dT_e \tag{33}$$

$$h\nu = \frac{1}{2}\pi\xi^2\gamma_S \left(T_{\text{hot}}^2 - T_0^2\right) \tag{34}$$

assuming 100% integrated electron-electron scattering efficiency [66]. There is no definitive formula for T_{hot} or ξ. However, for a 1550 nm incident photon, $T_{\text{hot}} = 100$ K would yield a hotspot size of $\xi = 94$ nm, that is consistent to the expectation [62, 64].

We then choose insulating boundary conditions at $x = 0$ and $x = 25$ μm in accordance with the dimensions of device B, solving Eqn. 1 numerically [46] in one dimension for each E-Ph scattering regime. The results are shown in Supplementary Figure 13A. Depending on the distance between the hotspot and the detector, the timing and magnitude of the initial temperature peak observed will be different. But after a certain amount of time, the entire graphene flake will thermalize to a uniform T_e, after which the spatial profile of T_e will be identical regardless of where on the graphene it is measured from. The timescale at which uniform temperature will be reached depends on the size of the device. For a 25 μm flake of graphene, it is on the order of 100 ps.

We can take the results of these simulations evaluated at one end of a 25 μm graphene heterostructure and integrate the time-dependent T_e to calculate the simulated detection efficiency η using Eqn. 31. Using an I_c of 3.38 μA and an I_b of 2.8 μA to simulate the conditions seen in Figure 2, with an integration time of 100 ns, we calculate η as a function of hotspot position for the two E-Ph coupling regimes of interest. The results, plotted in Supplementary Figure 13B, show that η may decrease with increasing distance away from the detector for $\delta = 3$. However, for $\delta = 4$, there is no appreciable reduction in η over 25 μm. The reason for this lies in the temperature at which the graphene reaches a uniform distribution of electronic heat. In the $\delta = 4$ case, after the graphene reaches a uniform temperature, it remains at a temperature substantially above $\Delta U/k_B$ for a sufficiently long amount of time that, regardless of the size of the initial peak during the first 0.1 ns, the junction will have 100% probability of switching. In contrast, the uniform temperature reached in the $\delta = 3$ case is lower, and remains elevated for less time, thermalizing to base temperature before 10 ns have elapsed. As such, whether the detector experiences elevated temperatures during the first 0.1 ns after photon absorption has a significant effect on the detection efficiency, causing a distance dependence in η.

1-D thermal modeling is not sufficient to definitively show that the devices measured in this paper follow the E-Ph coupling characteristic of the clean regime ($\delta = 4$) — the presence of multiple different E-Ph coupling mechanisms affecting the graphene cannot be ruled out. However, these calculations demonstrate that the independence of η as a function of distance from the JJ as seen

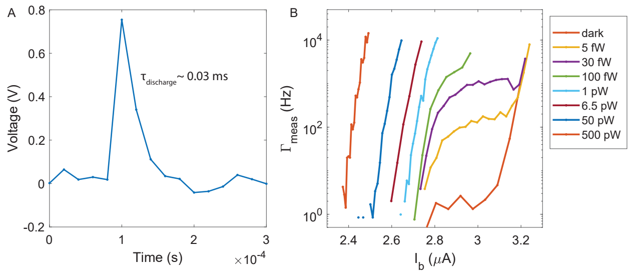

Supplementary Figure 14. **Measurement bandwidth limitations and photon flux limitations of the device (A)** A single photon event recorded on the digital to analog converter (DAC). The sharp spike in voltage is due to the junction switching from a photon detection. The decay of the voltage happens over $\tau_{\text{discharge}} \sim 0.03$ ms corresponding to the 30 kHz cut off of the filter. **(B)** Γ_{meas} as a function of I_b as the photon flux rate is increased. We find the device can support a photon flux of nearly 30 fW (corresponding to $\sim$ 1500 photons/second absorbed into the graphene) before the self-switching regime moves inward in bias. We attribute this to correlation effects due to the filter.

in our device is consistent with a realistic model of heat propagation in graphene.

Supplementary Note 16: Measurement Bandwidth and Photon Flux Considerations

While the rise time of the device is near "instantaneous", the reset is limited by the cryogenic filters which are designed at a 30 kHz cut off frequency (see Supplementary Figure 14A for a time domain trace of a single photon switching event). These filters are external to the chip and are mounted directly onto the plates of the dilution refrigerator. Therefore, in this work, the measurement bandwidth is limited to be under 30 kHz.

Similarly, care must be taken to ensure that the absorbed photon flux rate is well below the filter's roll-off. As an example, the highest powers presented (1 fW) corresponds to a flux of 7800 photons/s, and (with an absorption of 0.6%) 50 absorbed photons/s placing the presented powers well under the cut-off frequency of the filter. To benchmark the maximum flux rate we measure Γ_{meas} at higher photon fluxes (see Supplementary Figure 14B). Here, powers as high as 30 fW (1500 photons/s absorbed by graphene) still show a single photon plateau without substantial correlation effects from discharge (as evidenced by the lack of movement in the self switching, high I_b regime). However, for 100 fW (5000 photons/s absorbed by graphene) the curves move towards lower values of I_b suggesting either the device itself has begun overheating or correlation effects from the RC discharge are becoming non-negligible. Given that the absorbed photon flux rate is comparable to the filter discharge, we ascribe this feature to correlation effects from the filter discharge.

The device itself can likely support much faster reset times in the case of a lighter filter and well-engineered stray capacitance. The thermal time constant has been measured to be 10 ns [27], which can serve as a lower bound of the reset time. To unlock the full potential, one would look to embed the junction within a microwave circuit as was done in Refs. [27, 34, 67] for continuous operation of microwave bolometry. In this case, the response time would be limited to the ringdown time of the resonator or the thermal time constant (whichever is longer).

I. Comparison to Other Platforms

In Table 6, we compare different leading 1550 nm superconducting detector platforms with our device. We find that our device is competitive with state-of-the-art SNSPDs for both η and dark count rate.

Supplementary Table 6. Comparison of Detector Platforms. Efficiencies marked with a *, denote system efficiency.

**Work**	**Platform**	**Efficiency**	**DCR**	**Timing Jitter**	**Count Rate**
This Work	GJJSPD	87%	1/s	2.7 ps [46]	1.5 kc/s
Ref. [68]	SNSPD	78%	158/s	50 ps	1.5 Gc/s
Ref. [69]	SNSPD	1%	100/s	68 ps	100 Mc/s
Ref. [70]	SNSPD	28%	6×10^{-6}/s	—	—
Ref. [71]	SNSPD	93%*	1/s	150 ps	25 Mc/s
Ref. [72]	mKID	0.38%	—	—	—
Ref. [73]	SPAD	50%*	20000/s	70 ps	1 Mc/s

[1] Z. H. Ni, H. M. Wang, J. Kasim, H. M. Fan, T. Yu, Y. H. Wu, Y. P. Feng, and Z. X. Shen, Nano Letters **7**, 2758–2763 (2007), ISSN 1530-6992, URL http://dx.doi.org/10.1021/nl071254m.

[2] Y. Y. Wang, R. X. Gao, Z. H. Ni, H. He, S. P. Guo, H. P. Yang, C. X. Cong, and T. Yu, Nanotechnology **23**, 495713 (2012), URL https://iopscience.iop.org/article/10.1088/0957-4484/23/49/495713.

[3] H. B. Heersche, P. Jarillo-Herrero, J. B. Oostinga, L. M. K. Vandersypen, and A. F. Morpurgo, Nature **446**, 56 (2007), URL http://www.nature.com/nature/journal/v446/n7131/full/nature05555.html.

[4] X. Du, I. Skachko, and E. Y. Andrei, Physical Review B **77** (2008), 0710.4984, URL http://link.aps.org/doi/10.1103/PhysRevB.77.184507.

[5] M. B. Shalom, M. J. Zhu, V. I. Fal'ko, A. Mishchenko, A. V. Kretinin, K. S. Novoselov, C. R. Woods, K. Watanabe, T. Taniguchi, A. K. Geim, et al., Nature Physics **12**, 318 (2015), ISSN 1745-2473, URL http://www.nature.com/doifinder/10.1038/nphys3592.

[6] G.-H. Lee, D. Jeong, J.-H. Choi, Y.-J. Doh, and H.-J. Lee, Physical Review Letters **107**, 146605 (2011), ISSN 0031-9007, URL http://link.aps.org/doi/10.1103/PhysRevLett.107.146605.

[7] I. V. Borzenets, F. Amet, C. T. Ke, A. W. Draelos, M. T. Wei, A. Seredinski, K. Watanabe, T. Taniguchi, Y. Bomze, M. Yamamoto, et al., Physical Review Letters **117**, 237002 (2016), ISSN 0031-9007, URL http://link.aps.org/doi/10.1103/PhysRevLett.117.237002.

[8] V. E. Calado, S. Goswami, G. Nanda, M. Diez, A. R. Akhmerov, K. Watanabe, T. Taniguchi, T. M. Klapwijk, and L. M. K. Vandersypen, Nature Nanotechnology **10**, 761 (2015), ISSN 1748-3387, URL http://www.nature.com/doifinder/10.1038/nnano.2015.156.

[9] I. Holzman and Y. Ivry, Advanced Quantum Technologies **2** (2019), ISSN 2511-9044, 1807.09060.

[10] A. Gaggero, S. J. Nejad, F. Marsili, F. Mattioli, R. Leoni, D. Bitauld, D. Sahin, G. J. Hamhuis, R. Nötzel, R. Sanjines, et al., Applied Physics Letters **97**, 151108 (2010), ISSN 0003-6951.

[11] D. V. Reddy, R. R. Nerem, S. W. Nam, R. P. Mirin, and V. B. Verma, Optica **7**, 1649 (2020).

[12] B. Korzh, Q.-Y. Zhao, J. P. Allmaras, S. Frasca, T. M. Autry, E. A. Bersin, A. D. Beyer, R. M. Briggs, B. Bumble, M. Colangelo, et al., Nature Photonics **14**, 250 (2020), ISSN 1749-4885, URL https://www.nature.com/articles/s41566-020-0589-x.

[13] K. Irwin and G. Hilton, Topics in Applied Physics pp. 63–150 (2005), ISSN 0303-4216.

[14] K. M. Patel, S. Withington, A. G. . Shard, D. J. Goldie, and C. N. Thomas, Journal of Applied Physics **135**, 224504 (2024), ISSN 0021-8979.

[15] X. Gan, K. F. Mak, Y. Gao, Y. You, F. Hatami, J. Hone, T. F. Heinz, and D. Englund, Nano Letters **12**, 5626 (2012), ISSN 1530-6984, URL http://pubs.acs.org/doi/abs/10.1021/nl302746n.

[16] M. Furchi, A. Urich, A. Pospischil, G. Lilley, K. Unterrainer, H. Detz, P. Klang, A. M. Andrews, W. Schrenk, G. Strasser, et al., Nano Letters **12**, 2773 (2012), ISSN 1530-6984, 1112.1549.

[17] B. Vasić and R. Gajić, Optics Letters **39**, 6253 (2014), ISSN 0146-9592.

[18] D. K. Efetov, R.-J. Shiue, Y. Gao, B. Skinner, E. D. Walsh, H. Choi, J. Zheng, C. Tan, G. Grosso, C. Peng, et al., Nature Nanotechnology **13**, 797 (2018), ISSN 1748-3387, URL https://www.nature.com/articles/s41565-018-0169-0.

[19] X.-H. Deng, J.-T. Liu, J.-R. Yuan, Q.-H. Liao, and N.-H. Liu, Europhysics Letters **109**, 27002 (2015), URL https://dx.doi.org/10.1209/0295-5075/109/27002.

[20] B. Sensale-Rodriguez, R. Yan, M. M. Kelly, T. Fang, K. Tahy, W. S. Hwang, D. Jena, L. Liu, and H. G. Xing, Nature Communications **3**, 780 (2012), ISSN 2041-1723, URL https://doi.org/10.1038/ncomms1787.

[21] J. D. Mehew, R. L. Merino, H. Ishizuka, A. Block, J. D. Mérida, A. D. Carlón, K. Watanabe, T. Taniguchi, L. S. Levitov, D. K. Efetov, et al., Science Advances **10**, eadj1361 (2024), https://www.science.org/doi/pdf/10.1126/sciadv.adj1361, URL https://www.science.org/doi/abs/10.1126/sciadv.adj1361.

[22] B. Saleh and M. Teich, *Fundamentals of Photonics*, vol. 1 of *Wiley Series in Pure and Applied Optics* (2019).

[23] E. Marom, B. Chen, and O. G. Ramer, Optical Engineering **18**, 180179 (1979), URL https://doi.org/10.1117/12.7972325.

[24] H. Urey, Appl. Opt. **43**, 620 (2004), URL https://opg.optica.org/ao/abstract.cfm?URI=ao-43-3-620.

[25] E. D. Walsh, W. Jung, G.-H. Lee, D. K. Efetov, B.-I. Wu, K. F. Huang, T. A. Ohki, T. Taniguchi, K. Watanabe, P. Kim, et al., Science **372**, 409 (2021), URL https://science.sciencemag.org/content/372/6540/409.

[26] E. D. Walsh, Ph.D. thesis (2020).

[27] G.-H. Lee, D. K. Efetov, W. Jung, L. Ranzani, E. D. Walsh, T. A. Ohki, T. Taniguchi, K. Watanabe, P. Kim, D. Englund, et al., Nature **586**, 42 (2020), ISSN 0028-0836, URL https://www.nature.com/articles/s41586-020-2752-4.

[28] E. D. Walsh, D. K. Efetov, G.-H. Lee, M. Heuck, J. Crossno, T. A. Ohki, P. Kim, D. Englund, and K. C. Fong, Physical Review Applied **8**, 024022 (2017), ISSN 2331-7019, URL https://link.aps.org/doi/10.1103/PhysRevApplied.8.024022.

[29] J. Yan, M.-H. Kim, J. A. Elle, A. B. Sushkov, G. S. Jenkins, H. M. Milchberg, M. S. Fuhrer, and H. D. Drew, Nature Nanotechnology **7**, 472 (2012), URL http://www.nature.com/nnano/journal/v7/n7/full/nnano.2012.88.html.

[30] K. C. Fong and K. Schwab, Physical Review X **2**, 031006 (2012).

[31] H. Vora, P. Kumaravadivel, B. Nielsen, and X. Du, Applied Physics Letters **100**, 153507 (2012), URL http://scitation.aip.org/content/aip/journal/apl/100/15/10.1063/1.3703117.

[32] X. Du, D. E. Prober, H. Vora, and C. B. Mckitterick, Graphene and 2D Materials **1** (2014).

[33] A. E. Fatimy, R. L. Myers-Ward, A. K. Boyd, K. M. Daniels, D. K. Gaskill, and P. Barbara, Nature Nanotechnology **11**, 335 (2016), ISSN 1748-3387, URL https://www.nature.com/articles/nnano.2015.303.

[34] R. Kokkoniemi, J. P. Girard, D. Hazra, A. Laitinen, J. Govenius, R. E. Lake, I. Sallinen, V. Vesterinen, M. Partanen, J. Y. Tan, et al., Nature **586**, 47 (2020), ISSN 0028-0836, URL https://www.nature.com/articles/s41586-020-2753-3.

[35] X. Cai, A. B. Sushkov, R. J. Suess, M. M. Jadidi, G. S. Jenkins, L. O. Nyakiti, R. L. Myers-Ward, S. Li, J. Yan, D. K. Gaskill, et al., Nature Nanotechnology **9**, 814 (2014), ISSN 1748-3387, URL https://www.nature.com/articles/nnano.2014.182.

[36] A. Blaikie, D. Miller, and B. J. Alemán, Nature Communications **10**, 1 (2019), ISSN 2041-1723, URL https://www.nature.com/articles/s41467-019-12562-2.

[37] G. Skoblin, J. Sun, and A. Yurgens, Applied Physics Letters **112**, 063501 (2018), ISSN 0003-6951, URL http://aip.scitation.org/doi/10.1063/1.5009629.

[38] S. Yuan, R. Yu, C. Ma, B. Deng, Q. Guo, X. Chen, C. Li, C. Chen, K. Watanabe, T. Taniguchi, et al., ACS Photonics **7**, 1206 (2020), ISSN 2330-4022.

[39] Q. Han, T. Gao, R. Zhang, Y. Chen, J. Chen, G. Liu, Y. Zhang, Z. Liu, X. Wu, and D. Yu, Scientific Reports **3**, 3533 (2013), URL http://www.nature.com/srep/2013/131218/srep03533/full/srep03533.html.

[40] J. Hrubý, O. Laguta, A. Sojka, L. S. Marie, R. Myers-Ward, D. K. Gaskill, A. E. Fatimy, P. Barbara, and P. Neugebauer, Applied Physics Letters **124**, 123505 (2024), ISSN 0003-6951.

[41] U. Sassi, R. Parret, S. Nanot, M. Bruna, S. Borini, S. Milana, D. D. Fazio, Z. Zhuang, E. Lidorikis, F. H. L. Koppens, et al., Nature Communications **8**, 14311 (2017), 1608.00569.

[42] G. D. Battista, K. C. Fong, A. Díez-Carlón, K. Watanabe, T. Taniguchi, and D. K. Efetov, Science Advances **10**, eadp3725 (2024).

[43] D. Halbertal, M. B. Shalom, A. Uri, K. Bagani, A. Y. Meltzer, I. Marcus, Y. Myasoedov, J. Birkbeck, L. S. Levitov, A. K. Geim, et al., Science **358**, 1303 (2017), ISSN 0036-8075, URL http://www.sciencemag.org/lookup/doi/10.1126/science.aan0877.

[44] M. H. Devoret, J. M. Martinis, and J. Clarke, Physical Review Letters **55**, 1908 (1985), ISSN 0031-9007, URL http://link.aps.org/doi/10.1103/PhysRevLett.55.1908.

[45] T. A. Fulton and L. N. Dunkleberger, Physical Review B **9**, 4760 (1974), ISSN 1098-0121, URL http://gateway.webofknowledge.com/gateway/Gateway.cgi?GWVersion=2&SrcAuth=mekentosj&SrcApp=Papers&DestLinkType=FullRecord&DestApp=WOS&KeyUT=A1974T236000017.

[46] C. Fried, B. J. Russell, E. G. Arnault, B. Huang, G.-H. Lee, D. Englund, E. A. Henriksen, and K. C. Fong, Physical Review Applied **21**, 014006 (2024), 2311.00228.

[47] E. H. Hwang and S. D. Sarma, Physical Review B **77**, 115449 (2008), URL https://link.aps.org/doi/10.1103/PhysRevB.77.115449.

[48] R. Bistritzer and A. H. Macdonald, Physical Review Letters **102**, 206410 (2009), URL https://link.aps.org/doi/10.1103/PhysRevLett.102.206410.

[49] A. C. Betz, F. Vialla, D. Brunel, C. Voisin, M. Picher, A. Cavanna, A. Madouri, G. Fève, J.-M. Berroir, B. Plaçais, et al., Physical Review Letters **109**, 056805 (2012), ISSN 0031-9007, 1203.2753.

[50] C. B. McKitterick, D. E. Prober, and M. J. Rooks, Physical Review B **93**, 075410 (2016), ISSN 2469-9950, URL http://link.aps.org/doi/10.1103/PhysRevB.93.075410.

[51] A. W. Draelos, A. Silverman, B. Eniwaye, E. G. Arnault, C. T. Ke, M. T. Wei, I. Vlassiouk, I. V. Borzenets, F. Amet, and G. Finkelstein, Physical Review B **99**, 125427 (2019), ISSN 2469-9950, 1812.11711.

[52] J. K. Viljas and T. T. Heikkila, Physical Review B **81**, 245404 (2010).

[53] J. C. W. Song and L. S. Levitov, Physical Review Letters **109**, 236602 (2012), ISSN 0031-9007, 1205.5257, URL http://xxx.lanl.gov/abs/1205.5257.

[54] M. W. Graham, S.-F. Shi, D. C. Ralph, J. Park, and P. L. McEuen, Nature Physics **9**, 103 (2013), ISSN 1745-2473, 1207.1249.

[55] W. Chen and A. A. Clerk, Physical Review B **86**, 670 (2012), ISSN 1098-0121, URL https://link.aps.org/doi/10.1103/PhysRevB.86.125443.

[56] K. C. Fong, E. E. Wollman, H. Ravi, W. Chen, A. A. Clerk, M. D. Shaw, H. G. Leduc, and K. C. Schwab, Physical Review X **3**, 041008 (2013), ISSN 2160-3308, URL https://link.aps.org/doi/10.1103/PhysRevX.3.041008.

[57] A. C. Betz, S. H. Jhang, E. Pallecchi, R. Ferreira, G. Fève, J.-M. Berroir, and B. Plaçais, Nature Physics **9**, 109 (2013), ISSN 1745-2473, 1210.6894.

[58] M. Massicotte, G. Soavi, A. Principi, and K.-J. Tielrooij, Nanoscale **13**, 8376 (2021), ISSN 2040-3364, 2105.08352.

[59] M. A. Aamir, J. N. Moore, X. Lu, P. Seifert, D. Englund, K. C. Fong, and D. K. Efetov, Nano Letters (2021), URL https://pubs.acs.org/doi/10.1021/acs.nanolett.1c01553.

[60] S. D. Sarma, S. Adam, E. H. Hwang, and E. Rossi, Reviews Of Modern Physics **83**, 407 (2011), ISSN 0034-6861, URL https://link.aps.org/doi/10.1103/RevModPhys.83.407.

[61] A. W. Draelos, M.-T. Wei, A. Seredinski, H. Li, Y. Mehta, K. Watanabe, T. Taniguchi, I. V. Borzenets, F. Amet, and G. Finkelstein, Nano Letters **19**, 1039 (2019), ISSN 1530-6984, URL http://pubs.acs.org/doi/10.1021/acs.nanolett.8b04330.

[62] J. C. W. Song, K. J. Tielrooij, F. H. L. Koppens, and L. S. Levitov, Physical Review B **87**, 155429 (2013), ISSN 1098-0121, URL http://link.aps.org/doi/10.1103/PhysRevB.87.155429.

[63] D. Brida, A. Tomadin, C. Manzoni, Y. J. Kim, A. Lombardo, S. Milana, R. R. Nair, K. S. Novoselov, A. C. Ferrari, G. Cerullo, et al., Nature Communications **4**, 1987 (2013), ISSN 2041-1723, URL http://www.nature.com/doifinder/10.1038/ncomms2987.

[64] A. Block, A. Principi, N. C. H. Hesp, A. W. Cummings, M. Liebel, K. Watanabe, T. Taniguchi, S. Roche, F. H. L. Koppens, N. F. v. Hulst, et al., Nature Nanotechnology **16**, 1195 (2021), ISSN 1748-3387, 2008.04189.

[65] B. A. Ruzicka, S. Wang, L. K. Werake, B. Weintrub, K. P. Loh, and H. Zhao, Physical Review B **82**, 195414 (2010), ISSN 1098-0121, 1005.3850.

[66] K. J. Tielrooij, J. C. W. Song, S. A. Jensen, A. Centeno, A. Pesquera, A. Z. Elorza, M. Bonn, L. S. Levitov, and F. H. L. Koppens, Nature Physics **9**, 248 (2013), URL http://www.nature.com.ezproxy.cul.columbia.edu/nphys/journal/v9/n4/full/nphys2564.html.

[67] R. Katti, H. Arora, O.-P. Saira, K. Watanabe, T. Taniguchi, K. C. Schwab, M. Roukes, and S. Nadj-Perge, arXiv (2022), 2208.13379.

[68] I. Craiciu, B. Korzh, A. D. Beyer, A. Mueller, J. P. Allmaras, L. Narváez, M. Spiropulu, B. Bumble, T. Lehner, E. E. Wollman, et al., Optica **10**, 183 (2023), ISSN 2334-2536, URL http://dx.doi.org/10.1364/OPTICA.478960.

[69] R. E. Warburton, A. McCarthy, A. M. Wallace, S. Hernandez-Marin, R. H. Hadfield, S. W. Nam, and G. S. Buller, Optics Letters **32**, 2266 (2007), ISSN 1539-4794, URL http://dx.doi.org/10.1364/OL.32.002266.

[70] J. Chiles, I. Charaev, R. Lasenby, M. Baryakhtar, J. Huang, A. Roshko, G. Burton, M. Colangelo, K. Van Tilburg, A. Arvanitaki, et al., Physical Review Letters **128** (2022), ISSN 1079-7114, URL http://dx.doi.org/10.1103/PhysRevLett.128.231802.

[71] F. Marsili, V. B. Verma, J. A. Stern, S. Harrington, A. E. Lita, T. Gerrits, I. Vayshenker, B. Baek, M. D. Shaw, R. P. Mirin, et al., Nature Photonics **7**, 210 (2013), ISSN 1749-4885, URL http://www.nature.com/doifinder/10.1038/nphoton.2013.13.

[72] W. Guo, X. Liu, Y. Wang, Q. Wei, L. F. Wei, J. Hubmayr, J. Fowler, J. Ullom, L. Vale, M. R. Vissers, et al., Applied Physics Letters **110**, 212601 (2017), ISSN 0003-6951, 1702.07993.

[73] F. Signorelli, F. Telesca, E. Conca, A. D. Frera, A. Ruggeri, A. Giudice, and A. Tosi, in *2021 IEEE International Electron Devices Meeting (IEDM)* (IEEE, 2021), pp. 20.3.1–20.3.4, URL http://dx.doi.org/10.1109/IEDM19574.2021.9720559.
